# Patient radiation dose during angiography and embolization for abdominal hemorrhage: the influence of CT angiography, fluoroscopy system, patient and procedural variables

**DOI:** 10.1186/s42155-022-00284-4

**Published:** 2022-02-16

**Authors:** Conor McCaughey, Gerard M. Healy, Hanin Al Balushi, Patrice Maher, Jackie McCavana, Julie Lucey, Colin P. Cantwell

**Affiliations:** 1 St James Hospital, Dublin, Ireland; 2 Department of Radiology, St Vincent’s University Hospital, Dublin, Ireland; 3 School of Medicine, University College Dublin, Dublin, Ireland; 4 Department of Medical Physics and Clinical Engineering, St Vincent’s University Hospital, Dublin, Ireland

**Keywords:** Angiography, Abdominal: embolisation, Humans, Hemorrhage, Radiation dose, Gastrointestinal, Safety

## Abstract

**Background:**

Angiography and embolization (AE) is a lifesaving, high radiation dose procedure for treatment of abdominal arterial hemorrhage (AAH). Interventional radiologists have utilized pre-procedure CT angiography (CTA) and newer fluoroscopic systems in an attempt to reduce radiation dose and procedure time.

**Purpose:**

To study the factors contributing to the radiation dose of AE for AAH and to compare to the reference standard.

**Materials and methods:**

This retrospective single-centre observational cohort study identified 154 consecutive AE procedures in 138 patients (median age 65 years; interquartile range 54–77; 103 men) performed with a C-arm fluoroscopic system (Axiom Artis DTA or Axiom Artis Q (Siemens Healthineers)), between January 2010 and December 2017. Parameters analysed included: demographics, fluoroscopy system, bleeding location, body mass index (BMI), preprocedural CT, air kerma-area product (PKA), reference air kerma (K_a,r_), fluoroscopy time (FT) and the number of digital subtraction angiography (DSA) runs. Factors affecting dose were assessed using Mann–Whitney U, Kruskal–Wallis one-way ANOVA and linear regression.

**Results:**

Patients treated with the new angiographic system (NS) had a median PKA, median K_a,r_, Q3 PKA and Q3 K_a,r_ that were 74% (*p* < 0.0005), 66%(p < 0.0005), 55% and 52% lower respectively than those treated with the old system (OS). This dose reduction was consistent for each bleeding location (upper GI, Lower GI and extraluminal). There was no difference in PKA (*p* = 0.452), K_a,r_ (*p* = 0.974) or FT (*p* = 0.179), between those who did (*n* = 137) or did not (*n* = 17) undergo pre-procedure CTA. Other factors significantly influencing radiation dose were: patient BMI and number of DSA runs. A multivariate model containing these variables accounts for 15.2% of the variance in K_a,r_ (*p* < 0.005) and 45.9% of the variance of PKA (*p* < 0.005).

**Conclusion:**

Radiation dose for AE in AAH is significantly reduced by new fluoroscopic technology. Higher patient body mass index is an independent key parameter affecting patient dose. Radiation dose was not influenced by haemorrhage site or performance of pre-procedure CTA.

## Introduction

Abdominal arterial hemorrhage (AAH) is a common emergency that is associated with significant morbidity and mortality, with the annual incidence of upper gastrointestinal hemorrhage alone estimated between 61 and 78 per 100,000 (Mullady et al. 2020). Angiography and embolization (AE) is a lifesaving procedure commonly performed by interventional radiology to treat AAH. AE has good technical and clinical success (Hur et al. 2014; Loffroy et al. 2010), but it is performed under fluoroscopic guidance involving a high radiation dose (Mean reference air kerma greater than 1Gy) (Miller et al. 2003), which has implications for the patient, both in terms of short-term deterministic effects (e.g. skin burns (Balter et al. 2010)) and potential long term risk of stochastic effects such as malignancy (Beebe et al. 2016; Lin 2010).

In recent decades, patient radiation dose reduction has become a central component of radiology practice, embodied by the principle ‘As low as reasonably achievable’ (ALARA) (International Atomic Energy Agency (IAEA) 2018). However, interventional radiology had lagged behind diagnostic radiology in terms of publications on this topic (Hansmann et al. 2017). The reference study for interventional radiology radiation dose levels, the RAD-IR study, produced several publications between 2003 and 09 and these focused upon procedures performed in the United States between 1999 and 2002 (Miller et al. 2003; Miller et al. 2009). In the interval since the RAD-IR study, progressive technological development in fluoroscopic systems have occurred including x-ray energy optimisation and detector quantum efficiency improvements to reduce patient dose. There has been an increase in the use of preprocedural diagnostic computed tomography angiography (CTA) to improve detection and localization of the bleeding site and to reduce patient radiation dose. Two more recent studies from Bundy et al, including patients treated at a single institution in the United States between 2014 and 18 and Baumann et al, including patients treated at a single institution in Europe from 2011 to 14, concluded that radiation doses during fluoroscopic embolization procedures have fallen in the interval since RAD-IR, due to advances in technology and radiation protection practices (Bundy et al. 2018). These studies grouped procedures in different ways, for example Bundy reported the doses for all endovascular embolization procedures combined together (*n* = 188) ‘regardless of etiology or intent’, whereas RAD-IR and Baumann reported dose for gastrointestinal hemorrhage localization and treatment (*n* = 94 and 239 respectively), but did not stratify by bleeding site (upper GI vs lower GI) or include extra-luminal sites of abdominal hemorrhage (e.g., within abdominal solid organs, peritoneum, abdominal wall). These distinctions are important when individualising data for informed patient consent and departmental quality assurance.

We aim to study the factors contributing to the radiation dose of AE for AAH and compare radiation dose to the reference standard.

## Materials and methods

**Fig. 1:**
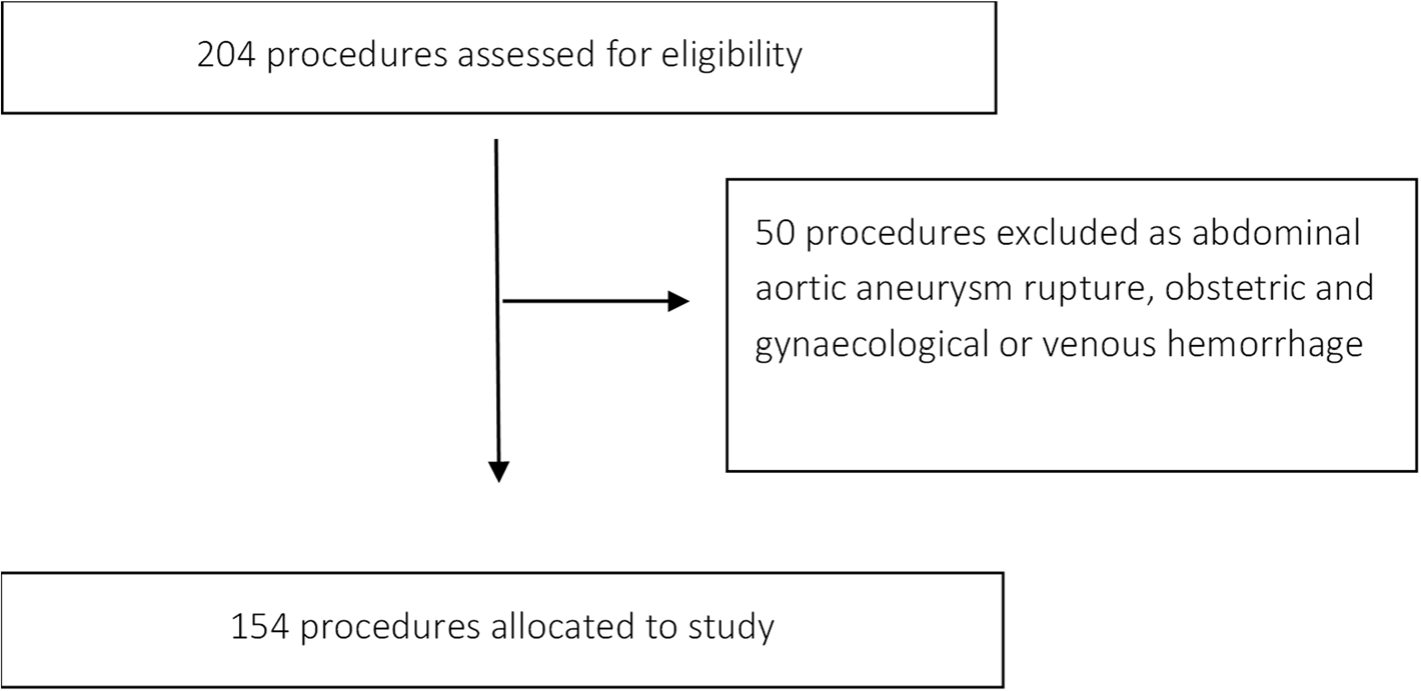
Flow diagram of patients with hemodynamic instability who underwent conventional angiography (CA) procedures for acute abdominal arterial hemorrhage during the study

Institutional review board approval was received for this retrospective study and the requirement for patient consent was waived. All patients who underwent AE for AAH with haemodynamic instability in our University Hospital between January 1st 2010 and the 31st of December 2017 were included. A list of AE procedures was obtained by a search of the radiology information system. This yielded a database of 204 consecutive procedures. Exclusion criteria were: abdominal aortic aneurysm rupture, obstetric and gynaecological or venous hemorrhage. After exclusions, this yielded a database of 154 procedures. See Fig. 1.

### Procedure

Procedures were performed by one of four fellowship trained interventional radiologists (with 15-, 9-, 9- and 5-years’ experience at the commencement of the study) under general anaesthetic or conscious sedation. Angiography was performed via femoral access. If active bleeding was identified on angiography or an abnormal vessel, selective branch embolization was performed using coils, liquid embolic or particles. If no active bleeding or abnormal vessel was seen, then empiric embolization of the arterial territory suspected as the source of hemorrhage was performed with gelfoam slurry at the operator’s discretion.

Procedures were performed on ceiling-suspended single plane angiography systems. Those procedures undertaken from the 1st January 2010 to the 31st of December 2013 were performed on Axiom Artis DTA (Siemens Healthineers), designated the old system (OS), while those procedures performed from the 1st of January 2014 to the 31st of December 2017 were performed on Axiom Artis Q (Siemens Healthineers), designated the new system (NS). The same radiation reduction and procedure protocols were used throughout the duration of the study. Standard methods of dose reduction were observed during every procedure; low pulse rate fluoroscopy, collimation, avoiding magnified views, low dose setting, anti-scatter grids and roadmap overlay technique.

The NS had a number of technical developments that aim to reduce radiation dose without affecting image quality: x-ray tube technology can deliver higher peak power, grid pulse technology facilitates shorter pulses of x-ray, a higher tube current with a higher prefilter produces a narrower spectrum of x-rays with less lower energy x-rays and a thicker scintillator leads to superior detector quantum efficiency (DQE). The NS also has post-processing algorithms for image sharpening and edge enhancement.

### Pre-procedure CTA

When deemed to be clinically indicated, pre-procedure abdominal CTA was performed on one of two identical 64-slice CT scanners (Somatom Cardiac CT, Siemens Healthineers, Erlangen, Germany). No oral contrast was administered. All images were reviewed by the operating vascular interventional consultant. Following acquisition of a non-contrast CT abdomen and pelvis range extended from the diaphragm to the symphysis pubis. 150 ml of low-osmolar iodinated contrast (340 mg/ml Iodine or higher concentration) was administered by power injector intravenously at 4 ml/ sec. Arterial-phase imaging was triggered using bolus tracking in the abdominal aorta at a density threshold of 125 Hounsfield units. The portal-venous-phase was acquired 25 s later and/or the delayed phase at 3 minutes after the arterial phase. The utilized CT tube parameter were between 100 and 140 kV and 180–586 mA. All CT scans were reconstructed and archived with contiguous thin sections of 1 mm thickness and routine acquisition and archiving of coronal and sagittal reconstructions. CT scans were primarily interpreted by an abdominal imaging fellowship trained attending. Active bleeding was defined as the detection of high-density contrast accumulation in the bowel lumen or an abdominal hematoma between the non-contrast and contrast-enhanced CTA phases. Acute vascular findings included the presence of active bleeding, pseudoaneurysm, truncated/irregular artery or arterio-venous fistula. A hematoma alone on CTA was defined as a negative CTA study.

### Data collection

The patients RISPACS record, clinical and electronic medical records were examined. Patient data was collected: age, sex, height, weight, body mass index (BMI) and BMI category using a computer-based abstraction form developed in a pilot study. For those patients whom height and weight data were incomplete (*n* = 122), BMI category was estimated from CT waist measurement using a previously described method (International Atomic Energy Agency (IAEA) 2018). We then validated this CT waist measurement method by comparing the results in the 32 patients where complete height and weight data was available.

Clinical data was recorded, including the bleeding location (upper gastrointestinal (GI), lower GI or extra-luminal, which included parenchymal, peritoneal or abdominal wall) and if a CTA was performed pre-procedure. Procedural and radiation dose data was recorded: fluoroscopy system used, procedure length (time from preparation of the patient’s groin until the time of the last image acquired), air kerma-area product (PKA, almost identical to dose area product), reference air kerma (K_a,r_, also known as cumulative dose), fluoroscopy time (FT) and the number of digital subtraction angiography (DSA) runs. If a patient had more than one AE procedure, each procedure was entered as a unique entry in the database.

### Statistical analysis

Baseline categorical variables were compared using a Pearson’s Chi square test for independent samples. The distribution of all scale variables was assessed with the Shapiro–Wilk test. Parametrically distributed variables were described using mean and 95% confidence intervals (CI) and compared using unpaired t tests. Non-parametric variables were described using median and interquartile range and were compared using Mann–Whitney U (two groups), or the Kruskal–Wallis (KW) one-way ANOVA (k groups). In cases where there was a significant difference between groups by ANOVA, post-hoc two-way comparisons between groups were performed using Mann–Whitney U test, with Bonferroni correction. For comparison with the RAD-IR study (the reference standard), mean and 95% CIs were calculated for non-parametric variables where applicable. For ordinal variables, count and percentage were presented and a Mann–Whitney U test was performed. Univariable (UVA) linear regression analysis was performed to identify variables which influenced patient radiation dose. All significant variables from UVA (*p* < 0.05) were entered into multivariate linear regression to create a model for patient radiation dose in the study cohort. All statistical computations were performed in SPSS statistics (version 25; IBM, New York, USA). The alpha value was set at 0.05.

## Results

138 patients were identified who underwent 154 AE procedures. The baseline characteristics of the entire cohort and the patients treated on the OS and NS are summarised in Table 1. A significantly higher proportion of patients with upper GI bleeding underwent AE using the new system (*n* = 29/38) compared to the OS (*n* = 9/38, *p* = 0.046). The groups were well matched in terms of gender and BMI. When the groups were stratified by bleeding location, those with a lower GI bleed were significantly older (median 76 yrs., IQR 60–81) compared to Upper GI bleed (median 64.5 yrs., IQR 53–71, *p* = 0.004) and extraluminal bleeding (median 65 yrs., IQR 48–76, *p* = 0.003).

**Table 1:** Baseline characteristics of patients compared between the old and new fluoroscopy system

Angiography Suite	OS (*N* = 61)	NS (*N* = 93)	*p*-value
Age^a^	70 (53–78)	65 (55–75)	0.835*
Gender			
Male	45 (74)	58 (62)	0.143
Female	16 (26)	35 (38)	
Bleeding Location			
Extraluminal	36 (59)	49 (53)	0.046
Upper gastrointestinal	9 (15)	29 (31)	
Lower gastrointestinal	16 (26)	15 (16)	
BMI Category			
Underweight (< 18.5)	6 (10)	4 (4)	0.369
Normal (18.5–25)	13 (21)	31 (33)	
Overweight (> 25- < 30)	22 (36)	34 (37)	
Obese I (30–35)	11 (18)	14 (15)	
Obese II (35–40)	9 (15)	10 (11)	

Except where indicated, data represent numbers of patients for categories and numbers in parentheses are percentages. a - data represented as median (Q1-Q3). *OS* old system (Axiom Artis DTA), *NS* new system (Axiom Artis Q)

**Fig. 2:**
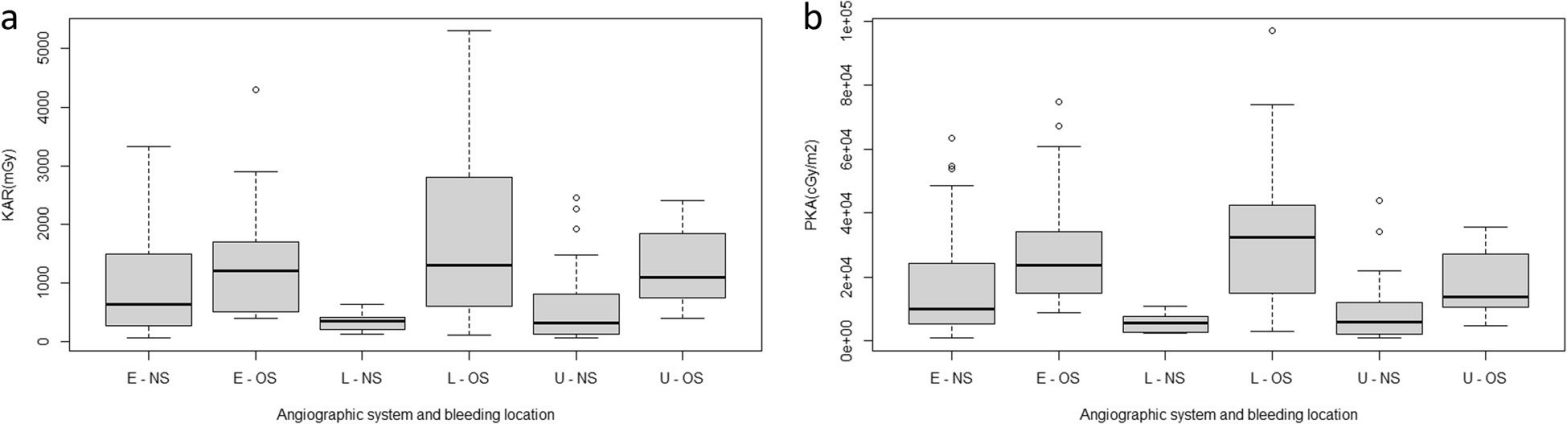
**a** Boxplot comparing reference air kerma (K_a,r_) between fluoroscopy systems, stratified by location. **b** Boxplot comparing air kerma-area product (PKA) between fluoroscopy systems, stratified by location. Values displayed as median and interquartile range. Outlying values denoted by circles. *E*=Extraluminal, *L*=lower GI and *U*=Upper *GI*; OS- Old system, *NS*- New System

Table 2 summarizes the procedure duration, mean FT, number of DSA runs, PKA, K_a,r_ stratified by bleeding location and compared between the machine types (OS, NS and bleeding location with OS and NS). Patients who underwent AE using the NS had a median PKA and K_a,r_ which was significantly lower than those using the OS (median PKA and K_a,r_ were 74% and 66% lower respectively; *p* < .005). The NS also demonstrated significantly lower radiation doses than the OS in patients with extraluminal and lower GI bleeding categories PKA and K_a,r_. In patients with upper GI bleeding, PKA was lower with the NS, but there was no significant different in K_a,r_. Boxplots comparing PKA and K_a,r_ by angiographic system and bleeding site are presented in Fig. 2.

**Table 2:** Comparison of procedure and radiation metrics, stratified by bleed location and angiographic system

Location	N	Duration (mins)	DSA runs	FT (seconds)	PKA (cGy.cm2)	Ka,r (mGy)
Extra-luminal						
OS	36	55 (38–74.5)	8 (6–10)	944.5 (661.5–1551.5)	23,694 (15011–35,681)	1200 (500–2000)
NS	49	65 (40–89)	7 (5–10)	1212 (738–1673)	8618 (3984–24,330)	635 (249–1668)
%					64	47
p					< 0.005	0.03
Upper GI						
OS	9	41 (30–56)	9 (6–10)	1378 (874–2395)	13,736 (10078–30,681)	950 (550–1850)
NS	29	54 (40–80)	8 (4–12)	885 (662–1749)	5511 (1545–11,686)	327 (114–847)
%					60	70
p					0.018	0.064
Lower GI						
OS	16	48 (27.5–64.5)	9 (6–11)	979.5 (622–1370)	28,788 (14573–41,471)	1200 (500–2450)
NS	15	56 (34–76)	7 (4–8)	747 (562–1778)	2906 (2435–7387)	344 (186–424)
%					90	71
p-					< 0.005	0.002
All locations						
OS	61	51 (35–71)	8 (6–10)	1009 (672–1524)	23,786 (14010–36,992)	1150 (500–2000)
NS	93	60 (40–86)	7 (4–9)	1083 (664–1744)	6227 (2435–16,568)	408 (183–953)
%					74	66
p					< 0.005	< 0.005

Unless otherwise stated, values are represented as the median, with the parenthesis containing the interquartile range

*FS* Fluoroscopy System, *OS* old system (Axiom Artis DTA), *NS* new system (Axiom Artis Q), FT - fluoroscopy time, *PKA* air kerma-area product, *K*_*a,r*_ reference air kerma, *%* Percentage change between systems

A comparison of procedure and radiation exposure metrics, stratified primarily by fluoroscopy system and secondly by bleed location is detailed in Table 3. Procedure time, DSA number, fluoroscopic time and K_a,r_ were not affected by the bleeding location on either fluoroscopy system. There was a significant difference in PKA medians between bleeding locations for the NS by KW-ANOVA (*p* = 0.025) but post hoc analysis found no significant between group differences (Table 4).

**Table 3:** Comparison of procedure and radiation exposure metrics, stratified by fluoroscopy system and bleed location

Location	N	Duration (mins)	DSA runs	FT (seconds)	PKA (cGy.cm2)	K_a,r_ (mGy)
OS						
Extraluminal	36	55 (38–74.5)	8 (6–10)	944.5 (661.5–1551.5)	23,694 (15011–5681)	1200 (500–2000)
Upper GI	9	41 (30–56)	9 (6–10)	1378 (874–2395)	13,736 (10078–0681)	950 (550–1850)
Lower GI	16	48 (27.5–64.5)	9 (6–11)	979.5 (622–1370)	28,788 (14573–41,471)	1200 (500–2450)
Subtotal	61	51 (35–71)	8 (6–10)	1009 (672–1524)	23,786 (14010–6992)	1150 (500–2000)
*p-value*		*0.367*	*0.768*	*0.534*	*0.349*	*0.794*
NS						
Extraluminal	49	65 (40–89)	7 (5–10)	1212 (738–1673)	8618 (3984–24,330)	635 (249–1668)
Upper GI	29	54 (40–80)	8 (4–12)	885 (622–1749)	5511 (1545–11,686)	327 (114–847)
Lower GI	15	56 (34–76)	7 (4–8)	747 (562–1778)	2906 (2435–7387)	344 (186–424)
Subtotal	93	60 (40–86)	7 (4–9)	1083 (664–1744)	6227 (2435–16,568)	408 (183–953)
*p-value*		*0.684*	*0.596*	*0.516*	0.025*	*0.055*

*OS* old system (Axiom Artis DTA), *NS* new system (Axiom Artis Q), *FT* fluoroscopy time, *PKA* air kerma-area product, *K*_*a,r*_ reference air kerma. *- There was a significant difference in PKA medians between bleeding locations for the NS by KW-ANOVA (p = 0.025) but post hoc analysis found no significant between group differences (Table 4)

**Table 4:** Adjusted *p*-values for the post hoc analysis of air kerma-area product for bleed locations using the new angiographic system

	Extraluminal	Upper GI
Upper GI	0.072 (W = 764)	–
Lower GI	0.072 (W = 341)	0.899 (W = 153)

Mann Whitney U test with Bonferroni Correction used for post-hoc analysis of difference in air-kerma product between bleeding locations for the new angiographic system. The Mann-Whitney statistic (W-value) is displayed in brackets

The AE cases preceded by a CTA scan (*n* = 137) did not vary significantly in their procedural parameters; FT (*p* = 0.179), DSA runs (*p* = 0.929), procedure duration (*p* = 0.094), or radiation dose (PKA, *p* = 0.452 and K_a,r_, *p* = 0.974), in comparison to those who did not have a pre-procedural CTA (*n* = 17).

The univariable and multivariable linear regression analysis of factors influencing radiation dose are detailed in Table 5. In multivariable analysis, the factors significantly influencing the PKA and K_a,r_ were number of the DSA runs, patient BMI and the fluoroscopy system used. Models containing these variables accounted for 45.9% and 15.2% of the variance in PKA and K_a,r_ respectively (*p* < 0.005 for both).

**Table 5:** Univariate and multivariate linear regression modelling of factors influencing radiation dose (*n* = 154)

	Univariate				Multivariate			
	R2	B	95% CI	p	R2	B	95% CI	p
Air-Kerma Product (PKA)					0. 459			< 0.005
Age	0.005	77.52	(−96)-251	0.379				
Duration	0.058	124	44–204	0.003		112	(−5.6)-231	0.062
FT	0.046	5.2	1.42–8.99	0.007		0.229	(−5.07)-5.53	0.932
DSA runs	0.158	1638	1028–2248	< 0.005		929	309–1548	0.004
Location	0.011	− 2307	(− 6306)-2050	0.197				
Indication	0.004	− 1752	(− 5766)-1692	0.449				
BMI	0.117	5701	3394–7970	0.001		5714	3632–7795	< 0.005
FS	0.199	−16,665	(−22,438)- (−10,800)	0.001		16,561	21,258–11,863	< 0.005
Reference Air Kerma (K_a,r_)					0.152			< 0.005
Age	0.005	6.8	(−10)-24	0.426				
Duration	0.013	5.7	(−2.63)-14.02	0.179				
FT	0.012	0.255	(−0.142)-0.653	0.206				
DSA runs	0.066	97.68	35.18–160.19	0.002		87	26.65–147.33	0.005
Location	0.006	− 171	(− 533)-189	0.329				
Indication	0.002	−117	(− 552)-317	0.595				
BMI	0.034	280	26–535	0.031		240	(−3.31)-483	0.053
FS	0.071	909	350–1467	0.002		822	1364–280	0.003

Table 5 displays the results of both the univariate and multivariate regression analysis of the patient and procedural factors influence on patient radiation dose, measured as both PKA (air kerma-area product) and K_a,r_ (reference air kerma). Only those factors that were found to have a significant impact on radiation dose during the univariate analysis were included in the multivariate analysis

*FT* Fluoroscopy Time, *FS* Fluoroscopy System

The results of the current study compared with those of the RAD-IR are summarised in Table 6. Of note, mean is used as the measure of central tendancy for this table in order to compare with that publication. Of the 94 patients included in part I of the ‘RAD-IR’ study who underwent fluoroscopic guided embolization for gastrointestinal bleeds, the mean PKA was found to be 34,757 cGy.cm^2^ (95%CI 2713–129,465 cGy.cm^2^), and the mean K_a,r_ was found to be 2367 mGy (95%CI 2037–2697 mGy). When comparing the mean radiation dose in our cohort with that of the RAD-IR study with the new system, an absolute 66% reduction was observed (Table 6).

**Table 6:** Comparison of procedure number, mean fluoroscopy time, air kerma-area product, reference air kerma between the RAD-IR study, old fluoroscopy system and new fluoroscopy system

	N	FT (min)		PKA (cGy.cm2)		K_a,r_ (mGy)	
		Mean (95% CI)	Range	Mean (95% CI)	Range	Mean (95% CI)	Range
RAD-IR	94	25.8 (22.2–29.5)	3.5–93.7	34,757 (30,599–38,915)	2713– 129,465	2367 (2037–2697)	105–7160
OS	61	19.6 (16.4–22.8)	1.33–61.9	28,540 (23,524-33,555)	138.5–96,876	1688 (1074-2302)	100–17,100
% Reduction compared to RAD-IR		24.0		17.9		28.7	
NS	93	20.8 (18.1–23.4)	0.6–62.4	11, 875 (8979-14,770)	261–63,118	778 (594–962)	52.8–3323
% Reduction compared to RAD-IR		19.4		65.8		67.1	

Note: The RAD-IR study used mean and not median values for FT, PKA and K_a,r_. Percentage reduction of PKA and K_a,r_ are calculated relative to the RAD-IR mean dose. *N* number, *OS* old system (Axiom Artis DTA), *NS* new system (Axiom Artis Q), *BMI* body mass index, *FT* fluoroscopy time, *PKA* air kerma-area product, *K*_*a,r*_ reference air kerma. *N/A* not available

**Fig. 3:**
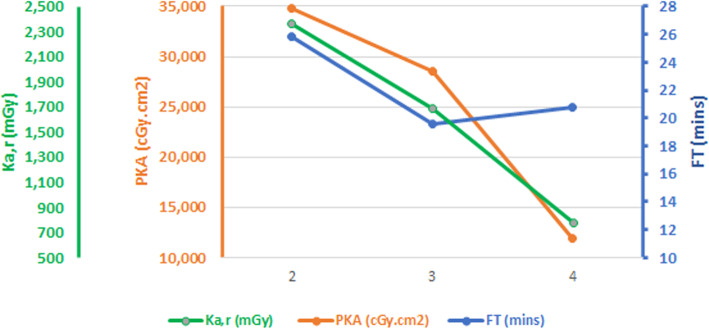
Graph comparing radiation metrics between the RAD-IR study, old system (OS) and new system (NS)

Subsequent analysis of the 94 patients in RAD-IR study led to a size-corrected dose reference levels (DRL) for gastrointestinal hemorrhage AE procedures of PKA of 31,915 cGy.cm^2^ (95%CI 26,916–36,082 cGy.cm^2^), and K_a,r_ 2056 mGy (95%CI 1797–2599 mGy). The same analysis suggested a DRL not-corrected for body habitus of PKA 52,000 cGy.cm^2^, K_a,r_ 3800 mGy, FT 35 min and 425 images (Miller et al. 2009). Our data would suggest the following DRLs (PKA, K_a,r_ and FT respectively) for patients undergoing AE for AAH, derived from the upper quartile values for the new system stratified by location: Extraluminal bleeding 24,300 cGy.cm^2^, 1668 mGy, 27.88 min, upper GI bleeding 11,686 cGy.cm^2^, 847 mGy, 29.15 min and lower GI bleeding 7387 cGy.cm^2^, 424 mGy, 29.63 min. When comparing the DRL in our lower gastrointestinal hemorrhage cohort on the NS with the RAD-IR DRL, our cohort PKA was 53% less, K_a,r_ 89% less and with comparable FT. A graph of the mean results of FT and mean K_a,r_ and PKA compared between RAD-IR and our study (OS, NS) shows reducing dose parameters and relatively stable FT, see Fig. 3.

## Discussion

The results of this study confirm that newer generation interventional radiology fluoroscopy systems, with more advanced technology, have the potential to significantly decrease the radiation being delivered to patients undergoing fluoroscopic guided procedures. The predictors of radiation exposure to the patients in our cohort were the fluoroscopic system used, the number of DSA runs and patient BMI, whereas the use of pre-procedural CTA or the location of bleeding did not impact on radiation dose.

Angiography equipment is the largest determinant of radiation dose for AE procedures. We have demonstrated a 66% reduction in K_a,r_ and 74% reduction in PKA at our centre with the introduction of a new angiographic system. The reference study in this field is RAD-IR (Miller et al. 2003; Miller et al. 2009) and judging by overlapping confidence intervals (Table 6), our OS demonstrated no significant difference compared to the RAD-IR doses, but the NS demonstrated a significant dose reduction, as measured by both outcome measures (65.8% reduction in PKA and 67.1% reduction in K_a,r_, Table 6). There is a clear trend of iterative improvement in radiation doses for this procedure over the past two decades (Fig. 1), however the fluoroscopy (Table 6) and procedure times (Fig. 3) are unchanged, suggesting similar procedure techniques and procedure difficulty across these studies. Our NS was also significantly lower than the recent European cohort of 139 patients who underwent AE for gastrointestinal bleeding between 2013 and 2014 (mean K_a,r_ 1342.9 mGy, 95% CI 1128.6–1557.2, compared to mean 778 mGy, 95% 594–962 from our NS), although they did not report PKA (Baumann et al. 2017). Therefore, the RAD-IR radiation doses and DRLs should no longer be considered the gold standard for this procedure, considering the changes in x-ray technology. This is supported by similar reductions in patient radiation dose in other recent studies of interventional procedures using new fluoroscopic technology, including uterine fibroid embolization (PKA reduction 77%) (Thomaere et al. 2018), TACE for hepatocellular carcinoma (PKA reduction 66%) (Schernthaner et al. 2015), bronchial artery embolization (PKA reduction 59%) (Spink et al. 2017) and cardiac catherization/angioplasty (PKA reduction 67%) (Buytaert et al. 2018).

CT angiography is sensitive in detecting and localising the source of hemorrhage. Pre-procedural CTA did not impact the patient radiation dose, fluoroscopy time or procedure duration during AE in this analysis. It has previously been shown that pre-procedure CTA does not influence the fluoroscopy time in lower GI hemorrhage angiography/embolization procedures (Jacovides et al. 2015), although we are not aware of any prior studies looking at the impact on radiation dose. There are other potential benefits of doing pre-embolization CTA, such as excluding patients who do not have active bleeding (Shukla et al. 2017; Foley et al. 2010), planning arterial access, planning procedural technique (Sommer et al. 2010), identifying the site (Jacovides et al. 2015) and cause of bleeding (for example, underlying malignancy) (Wells et al. 2018) and providing vital information for other services who may be involved in treating the patient (gastroenterology, surgery) (Jacovides et al. 2015). Hence, pre-procedure CTA will remain part of our local treatment algorithm for patients with suspected abdominal arterial haemorrhage.

Tabulating and publishing updated patient radiation doses for AE is relevant both for informed patient consent and departmental quality assurance. Studies have demonstrated that the majority of patients and physicians are either not aware or underestimate the potential risks associated with radiation in medical imaging (Lam et al. 2015), yet the majority of patients feel that anticipated procedure dose and associated radiation risks should be discussed with them as part of the consent process (Lam et al. 2015; Zener et al. 2018), even in the emergency setting (Takakuwa et al. 2010). Long fluoroscopic guided procedures, such as embolization, fall into the category of high dose radiology studies (> 1 mSv), for which a dedication discussion of radiation is advised during patient informed consent (Semelka et al. 2012). There is a considerable volume of misinformation in the general media about medical radiation, which can cause patient anxiety (Hendee and O'Connor 2012), but there is evidence to show that when a patient has accurate knowledge of the risks involved, along with the indication for the procedure/test, they are unlikely to decline (Zener et al. 2018; Takakuwa et al. 2010). Hence, there is a clear need for further education. Gathering procedure-specific data is essential to inform this process.

Information on the impact of BMI can further help to personalize the risks communicated to the patient, since radiology dose management for obese patients in IR has been highlighted as particularly important in guidelines (Zener et al. 2018). The positive correlation between BMI and radiation dose in fluoroscopy has been demonstrated previously in several studies, including in uterine fibroid (Lacayo et al. 2020), prostate artery (Barral et al. 2021) and visceral embolization (Baumann et al. 2017). In our study, for every increase in BMI category (for example from underweight to normal), a patient’s dose increased on average by 5714 cGy.cm2 (95%CI 3632–7795), as measured by PKA.

Quality assurance programs and regular audit are essential components of radiation dose optimization for any radiology department and they require accurate, relevant and up-to-date reference standards (dose references levels, DRL) from which to compare local performance (Tsapaki 2020). This is essential in order to identify equipment malfunction, incorrect protocols and/or deviations from normal practice by the users. This data may also be used in business cases for equipment upgrades. Pilot studies have shown that artificial intelligence algorithms could be useful in radiation quality assurance, however accurate training data with detailed labels (i.e., procedures specific radiation doses) are required in order train and validate such algorithms (Neri et al. 2019; Meineke et al. 2019).

This single-site retrospective study presents several limitations. The radiation doses described may not be generally applicable due to the impact of confounding variables including configuration and protocol differences between equipment, patient positioning, local practice patterns and operator experience. Additionally, as both systems were serially assessed, increased operator experience could represent a confounding factor in the decreased radiation exposure observed with the NS. However, the absence of observed significant differences in procedure metrics between both groups would suggest this was not a significant factor. The impact of patient anatomy and pathology was not considered and may affect the complexity of cases. The number and quality of images was neither recorded nor qualitatively assessed. Conclusions regarding the impact of pre-procedure CT on radiation exposure may be limited by the small number of patients who did not undergo pre-procedure CT (*n* = 17).

We cannot fully determine the significance of the reduction in radiation dose between RAD-IR and our cohort due to the absence of demographic and other details in the RAD-IR publication.

## Conclusion

Patient radiation dose during angiography and embolization in acute abdominal hemorrhage are significantly influenced by fluoroscopy technology, lower body mass index patients and the number of DSA runs performed. Site of hemorrhage does not affect radiation dose. Performance of a pre-procedure CTA may not impact procedure dose.

## Data Availability

The datasets used and subsequently analysed during the current study are available from the corresponding author on reasonable request.

## Abbreviations

AAH: Abdominal arterial hemorrhage
AE: Angiography and embolization
ANOVA: Analysis of variance
BMI: Body mass index
CI: Confidence interval
CTA: Computed Tomography Angiography
DSA: Digital subtraction angiography
DRL: Dose reference level
FT: Fluoroscopy time
K_a,r_: Reference air kerma
KW – ANOVA: Kruskal–Wallis (KW) one-way ANOVA
NS: New system
OS: Old System
PKA: Air kerma-area product
UVA: Univariable analysis
